# Endogenous calcitonin regulates lipid and glucose metabolism in diet-induced obesity mice

**DOI:** 10.1038/s41598-018-35369-5

**Published:** 2018-11-19

**Authors:** Misa Nakamura, Sachiko Nomura, Tadashi Yamakawa, Ryohei Kono, Akihiro Maeno, Takashi Ozaki, Akitoshi Ito, Toyonobu Uzawa, Hirotoshi Utsunomiya, Kennichi Kakudo

**Affiliations:** 1 Department of Rehabilitation, Osaka Kawasaki Rehabilitation University, 158 Mizuma, Kaizuka, Osaka 597-0104 Japan; 2 Department of Strategic Surveillance for Functional Food and Comprehensive Traditional Medicine, Wakayama Medical University, 811-1 Kimiidera, Wakayama, Wakayama 641–8509 Japan; 3 Department of Endocrinology and Diabetes, Yokohama City University Medical Center, 4-57 Urafunecho, Minamiku, Yokohama 232-0024 Japan; 4 Department of Medical Chemistry, Kansai Medical University, 2-5-1 Shin-machi, Hirakata, Osaka 573-1010 Japan; 5 Clinical Laboratory Department, Kinan Hospital, 46-70 Shinjo-cho, Tanabe, Wakayama 646-8588 Japan; 6 Laboratory for Pharmacology, Pharmaceuticals Research Center, Asahi Kasei Pharma Co. Ltd, 632-1 Mifuku, Izunokuni, Shizuoka 410-2321 Japan; 7 Medical Affairs Department, Asahi Kasei Pharma Co. Ltd, 1-1-2 Yurakucho, Chiyoda-ku, Tokyo 100-0006 Japan; 8 Department of Pathology, Nara Hospital, Kindai University Faculty of Medicine, 1248-1 Otoda-cho, Ikoma, Nara 630-0293 Japan

## Abstract

Calcitonin (CT) plays an important role in calcium homeostasis, and its precursor, proCT, is positively associated with the body mass index in the general human population. However, the physiological role of endogenous CT in the regulation of metabolism remains unclear. Knockout mice with gene-targeted deletion of exon 4 of *Calca* (CT KO) were generated by targeted modification in embryonic stem cells. Male mice were used in all experiments and were fed a slightly higher fat diet than the standard diet. The CT KO mice did not exhibit any abnormal findings in appearance, but exhibited weight loss from 15 months old, i.e., significantly decreased liver, adipose tissue, and kidney weights, compared with wild-type control mice. Furthermore, CT KO mice exhibited significantly decreased fat contents in the liver, lipid droplets in adipose tissues, serum glucose, and lipid levels, and significantly increased insulin sensitivity and serum adiponectin levels. CT significantly promoted 3T3-L1 adipocyte differentiation and suppressed adiponectin release. These results suggested that CT gene deletion prevents obesity, hyperglycemia, and hyperlipidemia in aged male mice. This is the first definitive evidence that CT may contribute to glucose and lipid metabolism in aged male mice, possibly via decreased adiponectin secretion from adipocytes.

## Introduction

**Figure 1:**
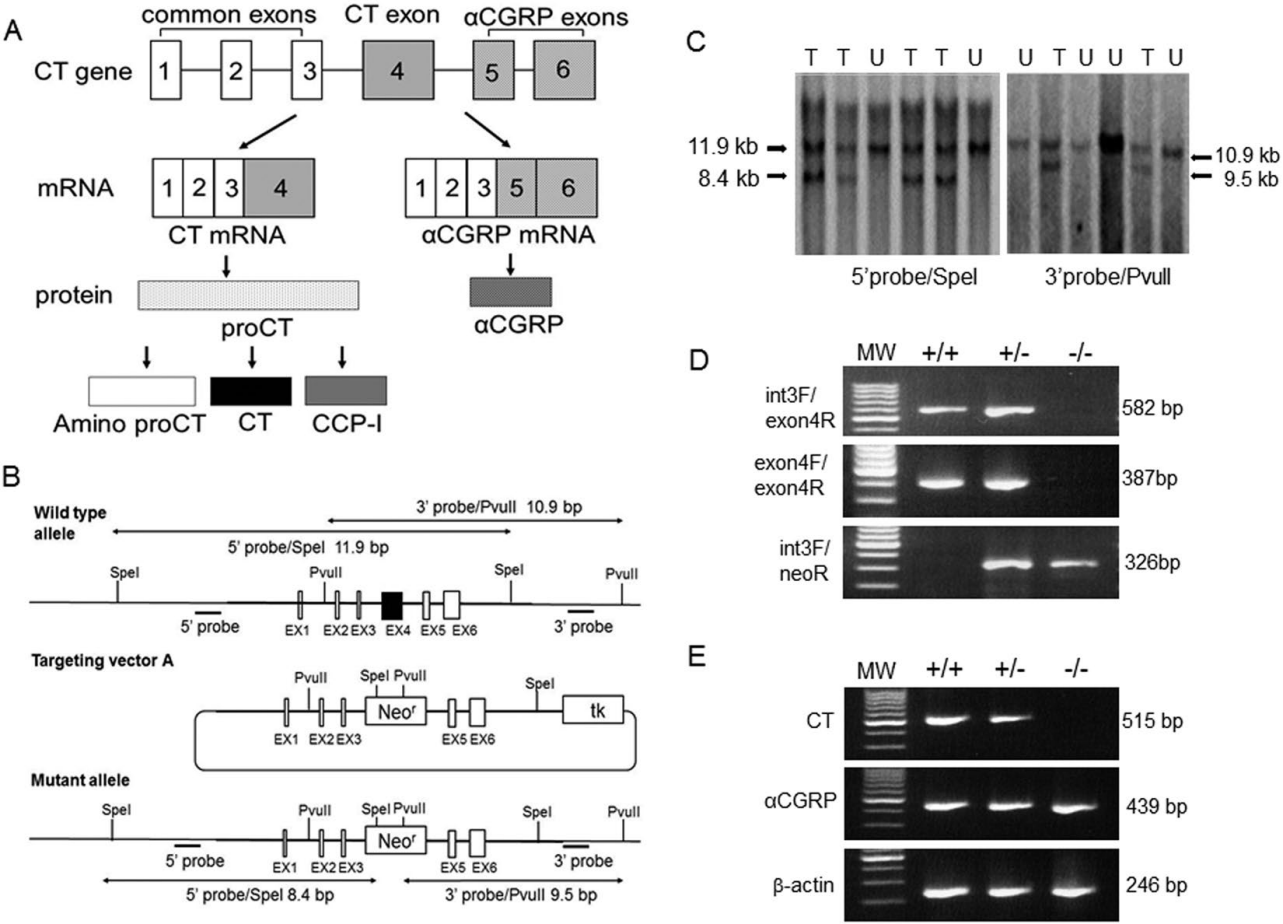
Production of CT KO mice. (**A**) Schematic representation of *Calca* tissue-specific alternative RNA processing and precursor peptide processing. CCP-I; CT carboxyl-terminus peptide-I. (**B**) Schematic of recombination of the targeting vector with *Calca*. Black bars indicate the general location of DNA probes used for Southern blotting. EX; exon, Neo^r^; neomycin resistance, tk; thymidine kinase. (**C**) Representative Southern blot analysis of SpeI or PvuII-digested DNA isolated from neomycin-resistant embryonic stem cells showing targeted (T) and untargeted (U) clones. A restriction map outlining the origin of individual bands is provided in B. (**D**) Identification of mouse genotype by PCR analysis of tail-derived DNA (see Methods). PCR products in +/+ show the presence of the normal WT allele; −/− shows the rearranged KO allele. Both alleles are detected in heterozygous animals (+/−). MW, 100-bp ladder molecular weight marker. E, RT-PCR analysis of mRNA isolated from the thyroid glands of WT or KO animals (see Methods). Primer pairs were exon 3F and exon 4R for detecting mRNAs for CT, exon 3F and exon 6 R for αCGRP, and β-actinF and β-actinR for β-actin.

Calcitonin (CT) is produced by thyroid C-cells and plays an important role in calcium homeostasis^1^. The gene encoding CT and calcitonin gene-related peptide α (αCGRP), known as *Calca*, is localized on chromosome 7 in mice^2^. The pre-mRNA from *Calca* contains six exons, and the mRNA of CT contains exons 1–4 and terminates after a polyadenylation site in exon 4. Another mRNA is also produced from this pre-mRNA in which exon 4 is skipped and exons 1–3, 5, and 6 are included. This pre-mRNA encodes the protein αCGRP^3^ in the central and peripheral nervous systems (Fig. 1A). The original product of exons 1–4 of *Calca* is preprocalcitonin, which is further processed to produce procalcitonin (proCT). The signal sequence at the amino terminus, together with its hydrophobic property, allows it to bind to the endoplasmic reticulum, where it is cleaved by an endopeptidase to generate proCT. The proCT in mice is composed of 136 amino acids and has a molecular weight of 17 kDa^4^. It is further processed to three peptides, aminopro CT, immature CT, and CT carboxyl-terminus peptide-I (designated catacalcin)^5^ (Fig. 1A). Practically all of the proCT formed in thyroid C-cells is converted to CT, so that no proCT enters into the circulation, and the level in healthy subjects is below the detection limit.

CT is primarily known as a regulator of osteoclast function, whereas αCGRP controls vascular tone and the activity of bone-forming osteoblasts^6^. Some reports have shown that αCGRP knock out (KO) mice are protected from diet-induced obesity via regulation of lipid metabolism and energy homeostasis^7–9^. Recently, Bartelt *et al*. reported that mice lacking *Calca*, which encodes CT and αCGRP, are protected from diet-induced obesity, nonalcoholic steatohepatitis, adipose tissue inflammation, and the mice display improved glucose tolerance^10^. Some reports have shown that a therapeutic dose of CT decreases levels of lipids containing cholesterol and triglyceride, improves energy and glucose homeostasis, and protects against obesity in rats^11–13^. Furthermore, higher CT levels in obese humans have been reported^14^. These results suggested that the CT family of peptides regulates lipid and glucose metabolism. However, the function of CT alone is not clear. Therefore, to clarify the role of CT in body weight regulation, we investigated phenotypic alterations in CT KO mice, in which *Calca* was modified to knock out CT expression while retaining normal αCGRP expression.

## Results

### Gene targeting

We created a targeting vector that disrupts exon 4 of *Calca* (Fig. 1B). Southern blotting demonstrated successfully targeted embryonic stem cells (Fig. 1C) that were implanted into female mice to generate chimeric founder mice, which were backcrossed onto the C57BL/6 background. Homozygous KO mice at exon 4 of *Calca* developed normally. Wild-type (WT) and KO animals did not differ in their general appearance. PCR analysis using primers that bind intron 3 (common to WT and KO), exon 4 (specific for WT), and neomycin (specific for KO) demonstrated the absence of *Calca* in homozygous −/− animals (Fig. 1D). A CT-deficient state with normal αCGRP expression was confirmed by RT-PCR analysis (Fig. 1E).

### Production and skeletal characterization of KO mice

Homozygous KO mice at exon 4 of *Calca* were born normally, and a similar number of pups per litter was born (average number of pups per litter, WT, 6.30 ± 0.58 pups; KO, 6.70 ± 0.58 pups). Pups of both genotypes were weaned around 20 days after birth. The length of the tibia at 3 months old was significantly different between WT and KO mice (WT, 17.90 ± 0.06 mm; KO, 17.40 ± 0.06 mm, n = 8 each, *p* < 0.0001).

### KO mice were protected from obesity in old age

**Figure 2:**
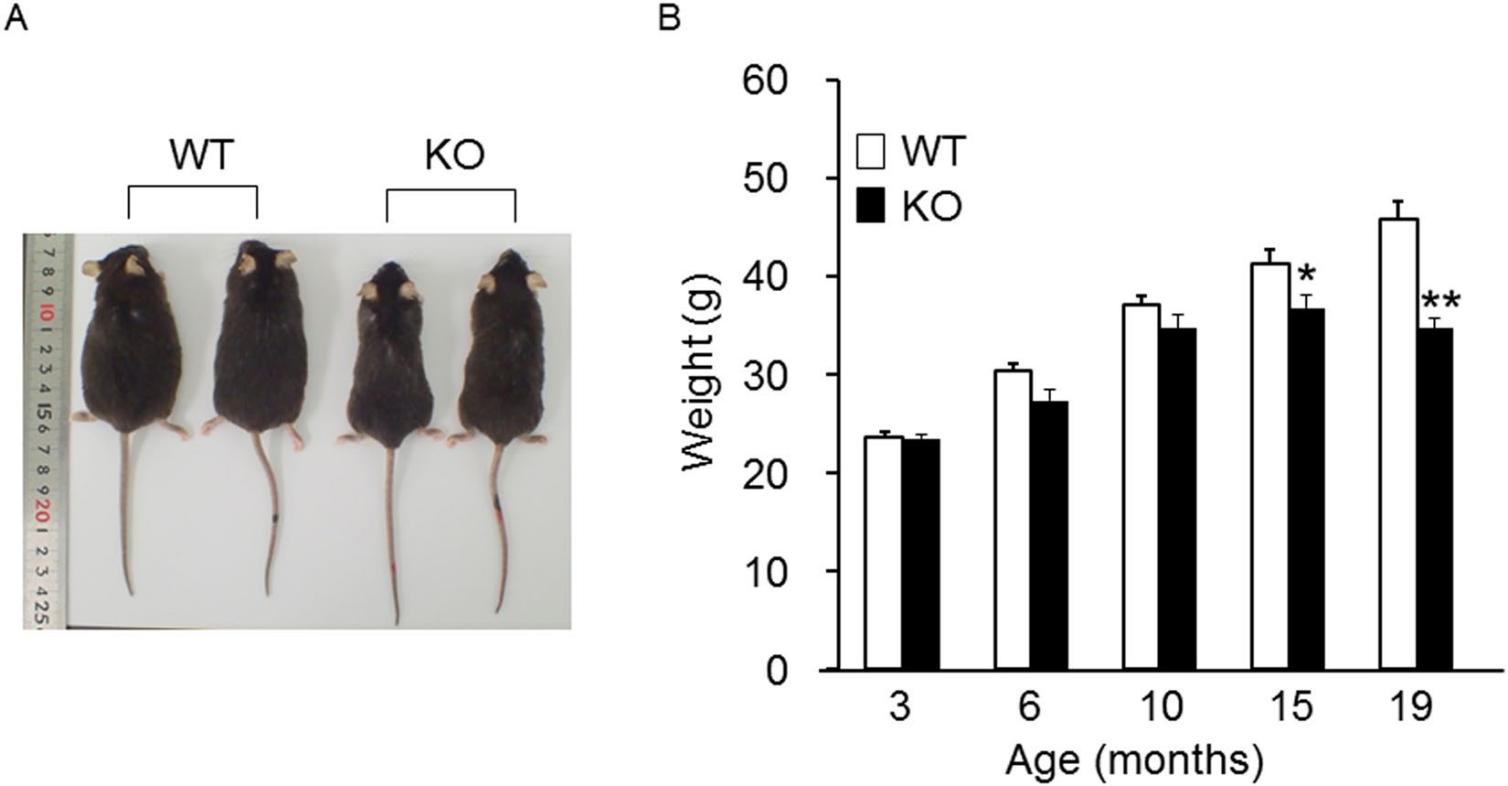
External appearances and body weights of WT and KO mice. (**A**) External appearances of WT and KO mice at 19 months of age. (**B**) Body weights of WT and KO mice at 3, 6, 10, 15, and 19 months of age (n = 8–17/group). WT body weight and KO body weight at each age were compared with the Student’s t test. *p < 0.05, **p < 0.01, vs. WT group.

Body weights up to the age of 10 months were not significantly different between WT and KO mice; however, body weights of KO mice at 15 and 19 months old were significantly lower than those of WT mice (Fig. 2A,B). We found no significant differences in daily energy intake between WT (12.64 ± 1.03 kcal) and KO mice at 12 months old (11.91 ± 1.03 kcal) (n = 8 each). These results suggested that CT KO mice were resistant to obesity in old age and that this was not related to an energy imbalance due to decreased caloric intake.

### KO mice showed decreased lipid accumulation in liver and adipocytes

**Figure 3:**
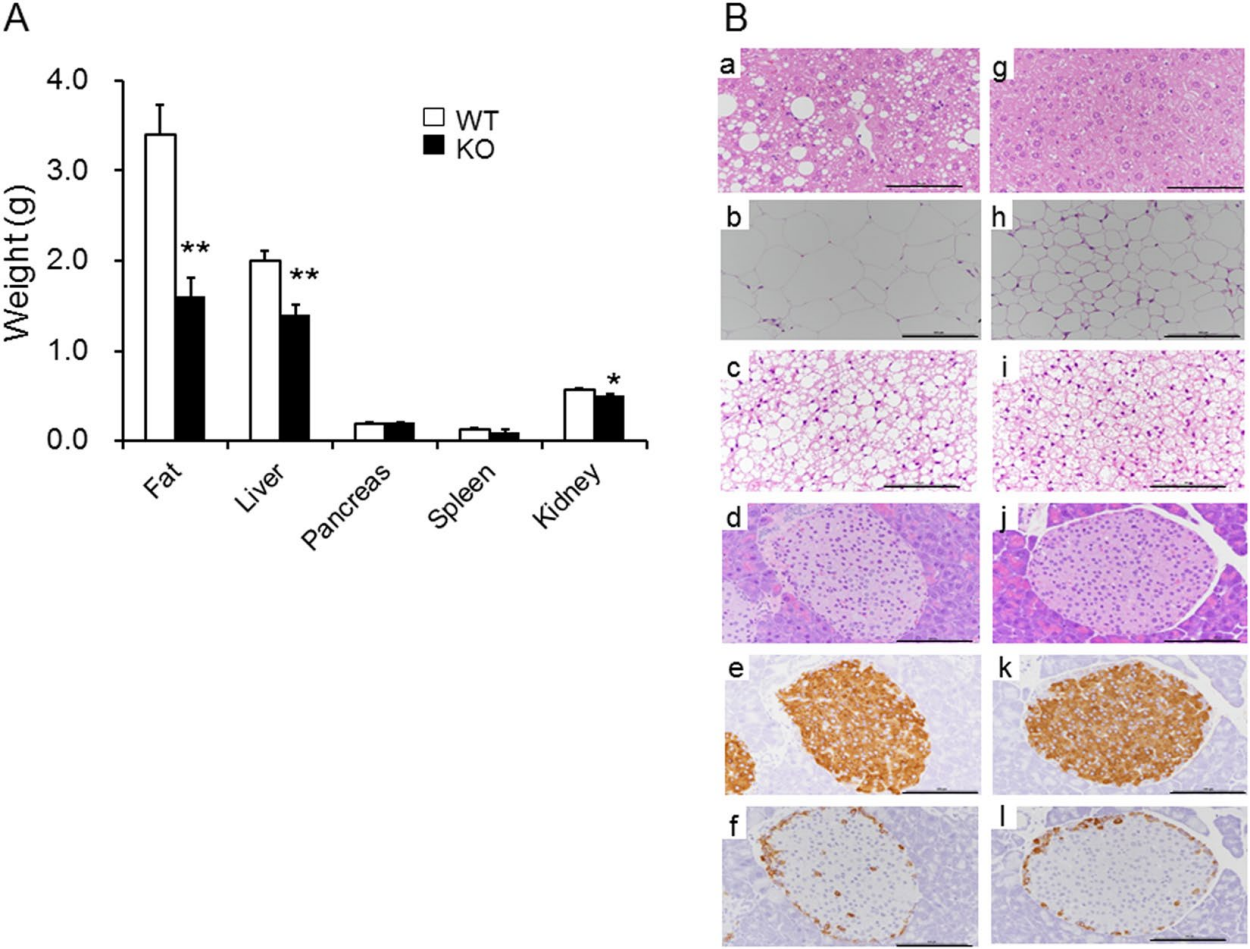
KO mice tended to show resistance to fatty liver. (**A**) The weights of organs at 19 months of age (n = 16–17/group). WT fat weight and KO fat weight were compared with the Welch’s t test. The weights of liver, pancreas, spleen, and kidney between WT and KO were compared with the Student’s t test. *p < 0.05, **p < 0.01 vs. WT group. (**B**) Hematoxylin and eosin staining of liver (a,g), intra-abdominal WAT (b,h), interscapular BAT (c,i), and islets (d,j) of WT (a–d) and KO (g–j) mice at 19 months of age. Immunostaining for β-cells (insulin staining) (e,k), and α-cells (glucagon staining) (f,l) of WT (a–f) and KO (g,h) mice at 19 months old. Scale bar: 100 µm.

No macroscopic differences were seen in liver, pancreas, spleen, or kidneys between WT and KO mice at autopsy examination. The weights of these organs at 19 months are shown in Fig. 3A. The results showed that the visceral fat, liver, and kidney weights were significantly decreased in KO compared to WT mice. The visceral fat content in WT mice was 2-fold greater than in KO mice. No differences were detected for pancreas and spleen weights between the two groups (Fig. 3A). The histological results of the liver, intra-abdominal white adipose tissue (WAT), interscapular brown adipose tissue (BAT), and pancreas of WT and KO are shown in Fig. 3B. In liver tissue, an increased size of lipid droplets in hepatocytes was observed in WT mice (Fig. 3B,a) and was significantly decreased in KO mice (Fig. 3B,g). The size of adipocytes in WAT was smaller in KO mice (Fig. 3B,h) than WT mice (Fig. 3B,b). Larger lipid droplets in BAT were observed in WT mice (Fig. 3B,c) than in KO mice (Fig. 3B,i). On the other hand, we observed no differences in the size of pancreatic islets or the numbers of insulin- and glucagon-positive cells (Fig. 3B,d–f and j–l).

### Decreased serum and liver lipids in KO mice

**Figure 4:**
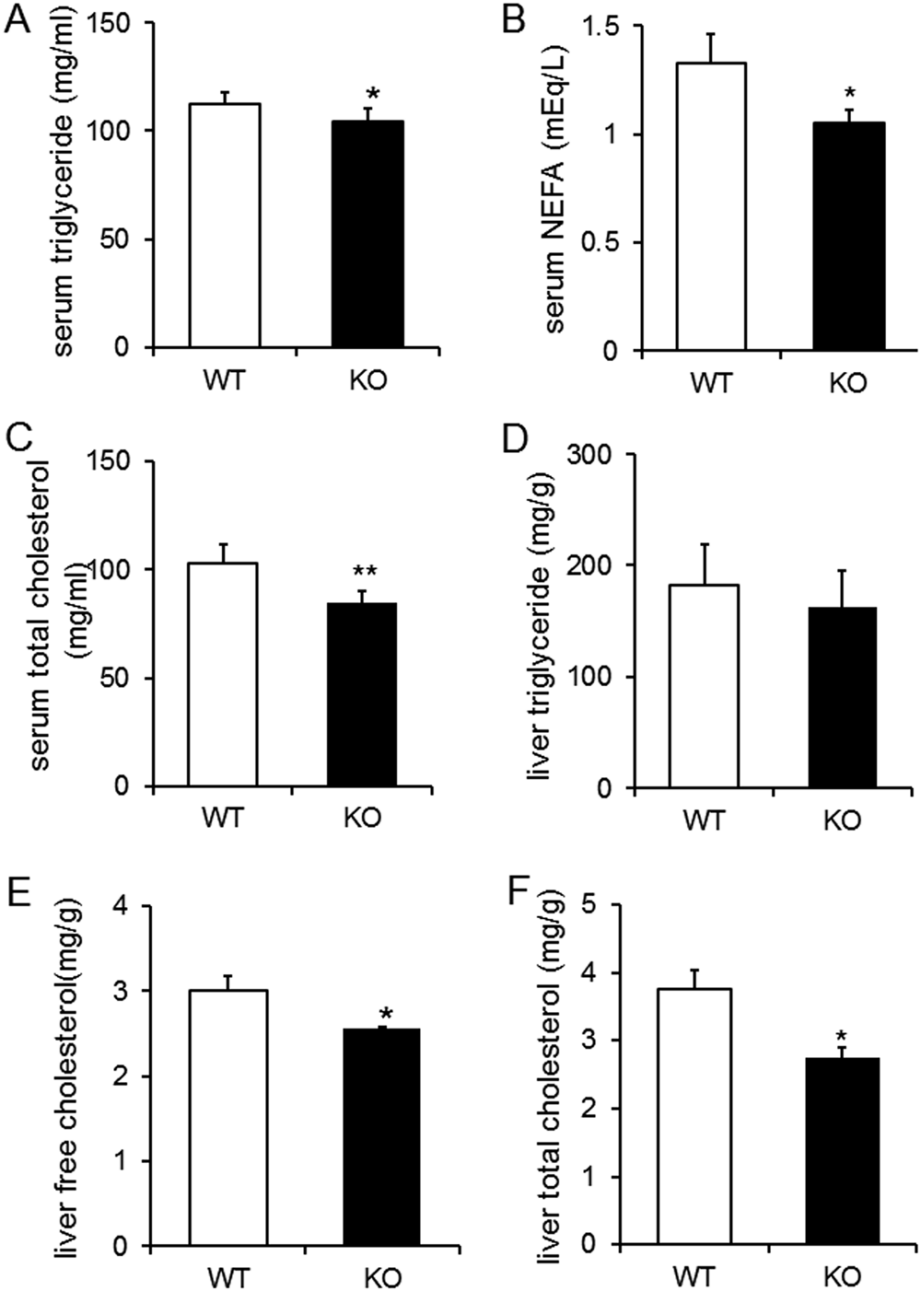
KO mice showed decreases in both serum and liver lipids. Serum triglyceride (**A**) serum NEFA (**B**) and serum total cholesterol (**C**) after 16 h of fasting at 14 months of age (n = 24–25/group). Liver triglyceride (**D**) liver free cholesterol (**E**) and liver total cholesterol (**F**) after 16 h of fasting at 17 months of age (n = 5/group). (**A,C,D,F**) were analyzed with the Student’s t test. B was analyzed with the Welch’s t test. E was analyzed with the Wilcoxon signed-rank test. *p < 0.05, **p < 0.01 vs. WT group.

Triglyceride, non-esterified fatty acid (NEFA), and total cholesterol in serum or liver were measured following fasting. The serum levels of triglyceride, NEFA, and total cholesterol were significantly lower in KO mice than WT mice (Fig. 4A–C). In liver tissue, the levels of free cholesterol and total cholesterol were significantly lower in KO mice than WT mice (Fig. 4E,F), which is in good agreement with our observation of decreased lipid droplets in hepatocytes (Fig. 3B,a,g). These results strongly suggest that CT deficiency in old CT KO mice had significant impacts on lipid metabolism in the liver and fatty tissue.

### Decreased serum glucose level in KO mice is related to insulin sensitivity

**Figure 5:**
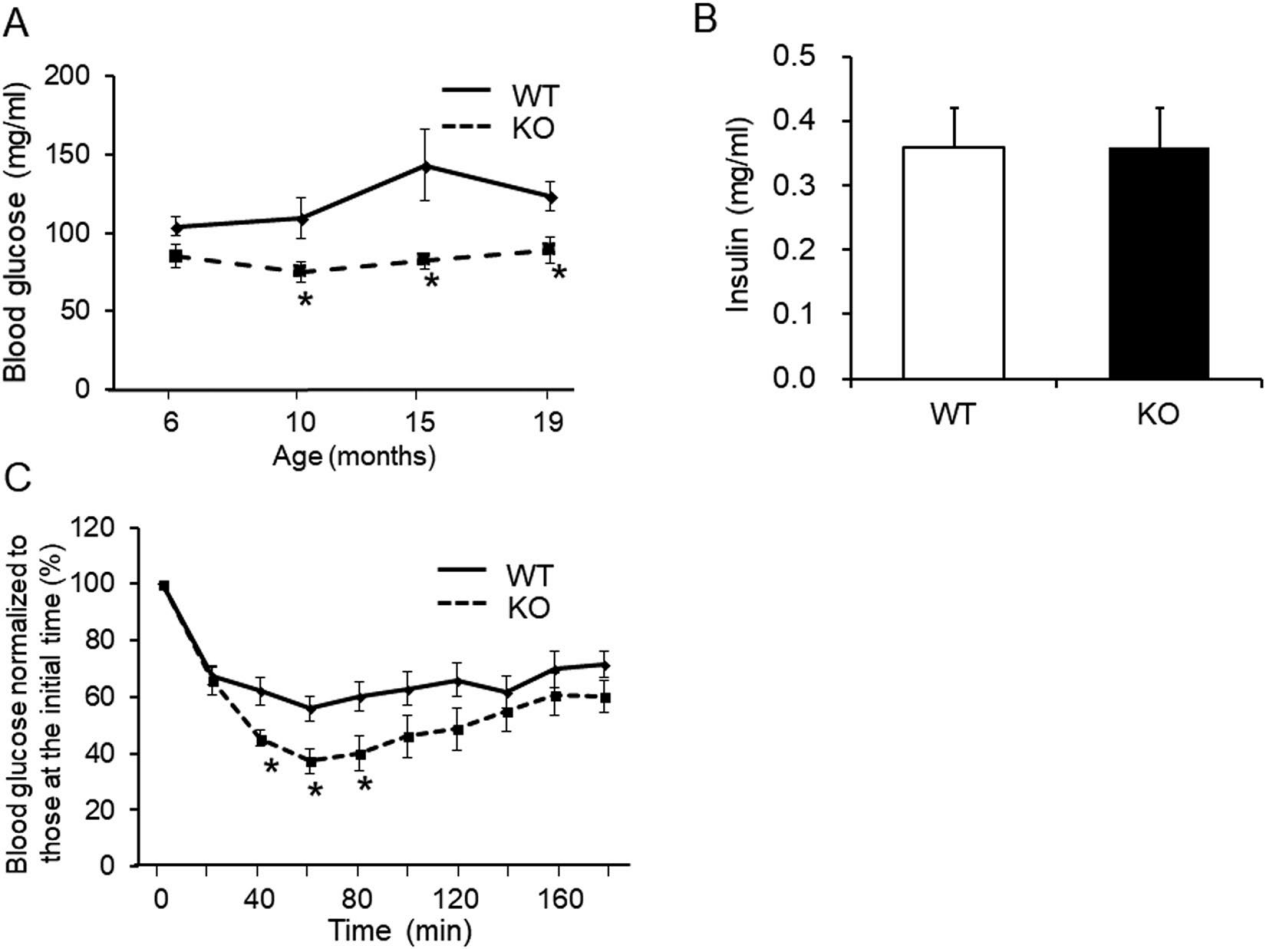
Decreases in serum glucose levels in KO mice were related to insulin sensitivity. (**A**) Blood glucose levels following 16 h of fasting in 19-month-old WT (solid line) and KO (dashed line) mice (n = 6/group). (**B**) Serum insulin levels following 16 h of fasting in 14-month-old mice (n = 7–8/group). (**C**) Blood glucose levels during the insulin tolerance test in 17-month-old WT (solid line) and KO (dashed line) mice following 3 h of fasting (n = 7–8/group). All results were analyzed with the Student’s t test. *p < 0.05 vs. WT group.

The CT KO mice showed significantly lower fasting blood glucose levels than WT mice at 10, 15, and 19 months of age (Fig. 5A). The serum insulin levels were not significantly different between the two groups (Fig. 5B). To evaluate whether insulin sensitivity was increased in KO mice, an intraperitoneal insulin tolerance test (ITT) was performed. Glucose levels were significantly lower in KO mice compared with WT at 40, 60, and 80 min after injection of insulin (Fig. 5C). These results suggest that KO mice may be resistant to high-fat diet-induced insulin resistance.

### KO mice have a higher level of serum adiponectin

**Figure 6:**
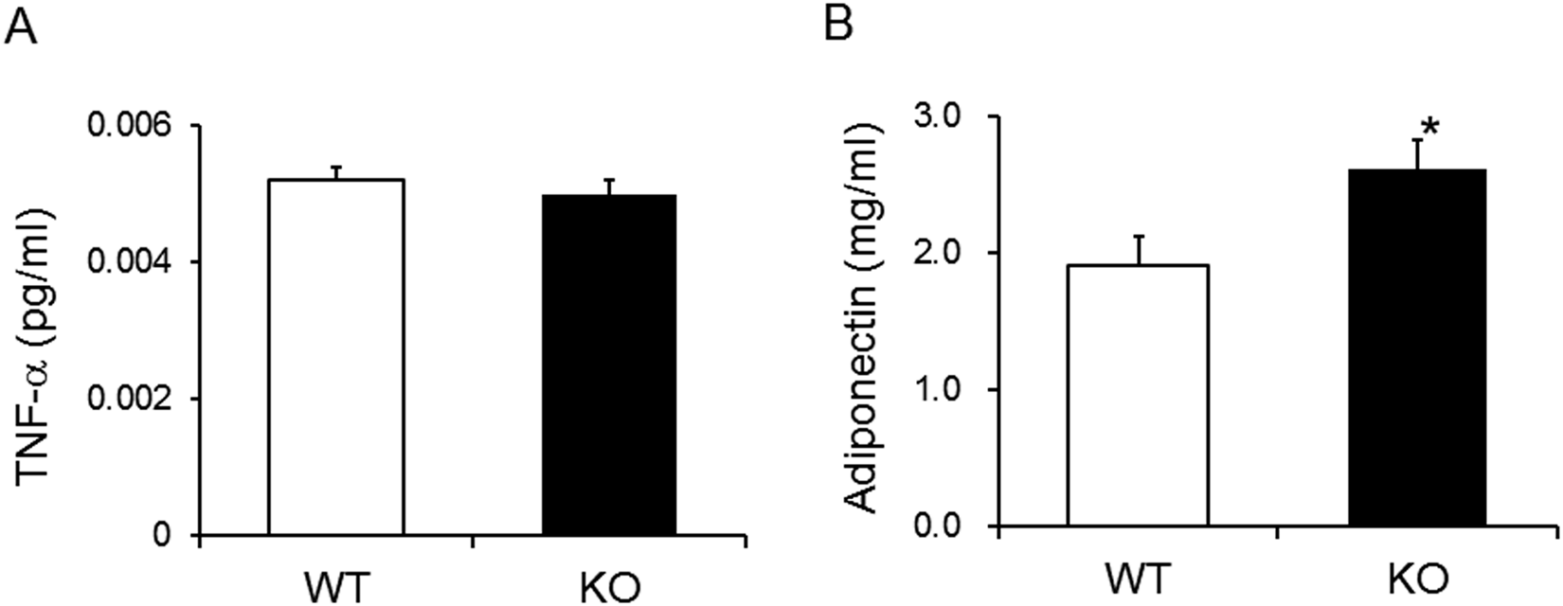
Serum adiponectin levels were higher in KO mice. Serum TNF-α (**A**) and adiponectin (**B**) following 16 h of fasting in 14-month-old mice (n = 7–8/group). A was analyzed with Student’s t test. B was analyzed with the Wilcoxon signed-rank test. *p < 0.05 vs. WT group.

Serum levels of adipokines (tumor necrosis factor (TNF)-α and adiponectin) were compared between WT and KO animals. TNF-α levels did not significantly differ between WT and KO mice (Fig. 6A). In contrast, adiponectin levels were significantly higher in KO mice than WT mice (Fig. 6B).

### CT increased lipid contents and decreased adiponectin production in 3T3-L1 cells

**Figure 7:**
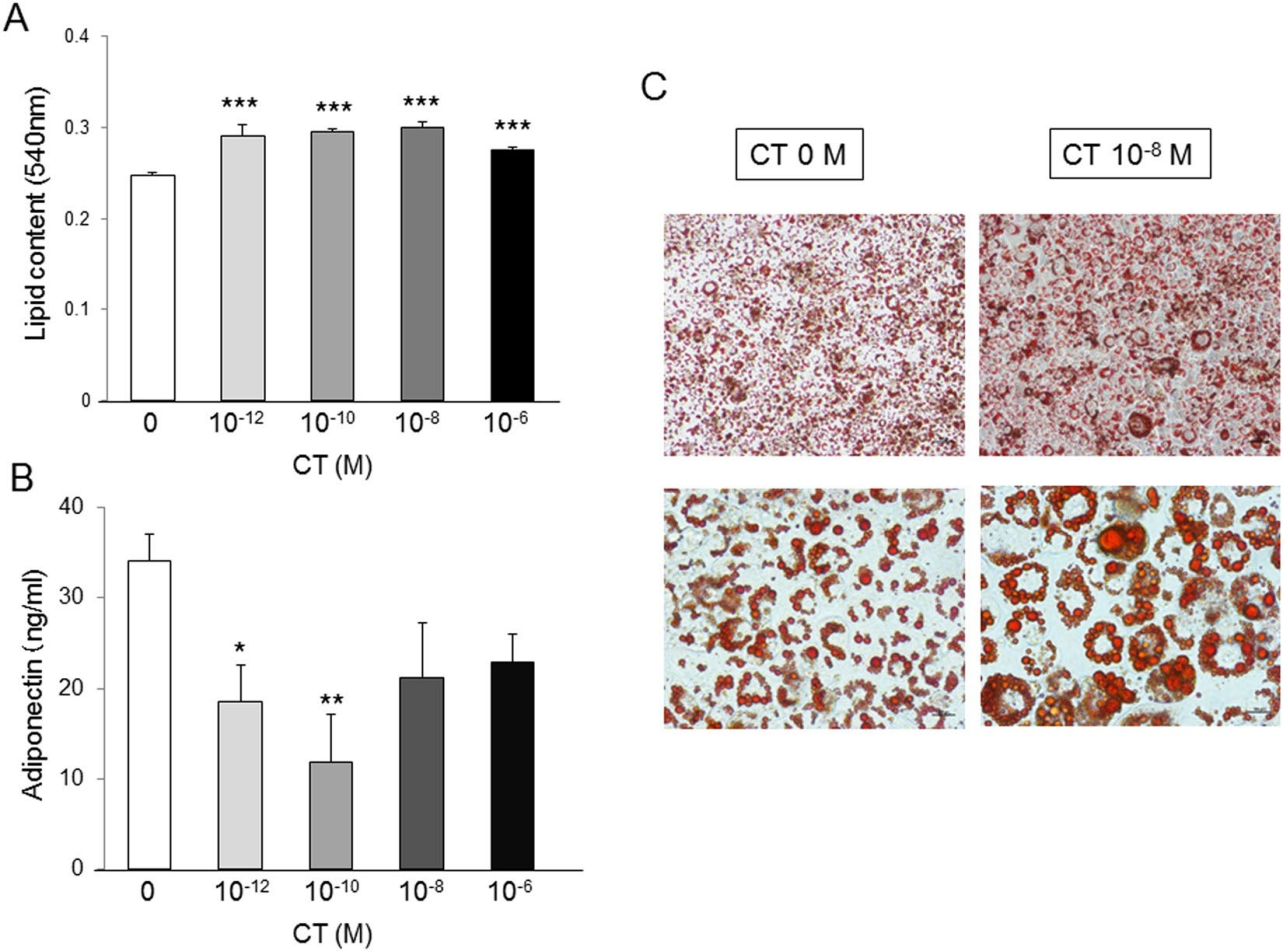
CT increased the lipid contents and decreased production of adiponectin in 3T3-L1 cells. (**A**) Lipid content in cells treated with different concentrations of CT during adipocyte differentiation for 4 days (n = 5/group). (**B**) Adiponectin levels in medium treated with different concentrations of CT during adipocyte differentiation for 4 days (n = 5/group). (**C**) Oil Red O staining of differentiated cells. Scale bar: 50 µm (top), 25 µm (bottom). (**A**,**B**) were analyzed with the Student’s t test. *p < 0.05, **p < 0.01, ***p < 0.0001 vs. control (0 M CT) group.

3T3-L1 cells were employed to clarify the effects of CT on adipocyte differentiation *in vitro*. Lipid contents in cells and adiponectin levels in the medium were measured following treatment with 10^−12^, 10^−10^, 10^−8^, and 10^−6^ M CT. Lipid contents were significantly higher in all CT groups than the control and were the highest in 10^−8^ M CT in particular (Fig. 7A,C). Adiponectin levels were significantly lower in the 10^−12^ M and 10^−10^ M CT groups than the control (Fig. 7B). These results suggested that CT increased lipid contents and decreased adiponectin production in 3T3-L1 cells.

## Discussion

Mice deficient in both CT and αCGRP are protected from diet-induced obesity and display improved glucose tolerance^10^. In this study, we generated CT KO mice, in which *Calca* was modified so that CT was not expressed but αCGRP expression remained. These KO mice showed weight loss in old age, and decreased liver and adipose tissue weights compared with the age-matched WT controls. Histological findings showed a decrease in fat accumulation in KO liver, WAT, and BAT, and quantitative results showed a decrease in fat accumulation in KO liver. Furthermore, decreases in adipocyte size, blood glucose level, and serum lipid levels, as well as increased serum adiponectin compared with the control were detected. These results may indicate that defects in CT in CT KO mice have favorable effects on diet-induced obesity by affecting lipid metabolism. On the other hand, renal lipid accumulation and glomerulosclerosis occur in diet-induced obese mice^15^. We inferred that the kidney weight of the KO was significantly lower than that in the WT due to the obesity-suppressing effect of KO of CT.

Walker *et al*. reported that αCGRP KO mice are protected from diet-induced obesity and show improved glucose metabolism and insulin sensitivity. Further, the αCGRP KO mice develop a range of parameters in keeping with a higher metabolic rate including higher body temperature and increased energy consumption, with the consequence of losing body weight^8^. However, the CT KO mice in the present study did not show increased daily energy intake, suggesting that the decreased body weight in KO mice in this study is independent of caloric intake.

Accumulation of lipids in the liver is associated with obesity, which can lead to nonalcoholic fatty liver disease and eventually nonalcoholic steatohepatitis^16^. Several reports support the physiological role of CT in modulating hepatocyte function. Specifically, CT receptors (CTRs) are present in the plasma membrane of rat hepatocytes^17^, and CT promotes hepatic fatty acid synthesis following administration in rats^18^. These studies support our findings of decreased fat contents in the CT KO mouse liver.

Like leptin and TNF-α,I adiponectin is an adipokine, which is specifically and abundantly expressed in adipose tissue and shows anti-inflammatory^19^, anti-atherogenic^20^, anti-hyperlipidemia^21^, and tissue-specific anti-diabetic^22^ properties. Furthermore, adiponectin increases with weight loss and is negatively correlated with body mass index^23–26^. In the present study, elevated adiponectin level, but not TNF-α was detected in KO mice. Cross-talk occurs between visceral adipose tissue and the liver. Visceral fat releases free fatty acids that are transported to the liver and may contribute to hepatic steatosis and nonalcoholic steatohepatitis^27,28^. Adiponectin antagonizes excess lipid storage in the liver and protects against inflammation and fibrosis^29^. Plasma adiponectin levels are also directly related to plasma HDL cholesterol levels and are inversely related to serum triglyceride levels^30^. *CTR* is expressed in adipocytes^31^. From these results, we postulated that the decrease in serum lipid concentrations and liver lipid accumulation in KO mice revealed in this study could be attributable to the high concentration of adiponectin.

Blood glucose levels were significantly decreased in KO compared to WT mice, and the serum insulin levels were not significantly different between the two groups. CT administration to normal and thyroid/parathyroidectomized rats causes increased blood glucose levels^32^. In the present study, differences in blood glucose levels during the ITT between control and KO mice were observed, suggesting that suppression of blood glucose levels in KO mice is attributable to increased insulin sensitivity. Adiponectin is an insulin-sensitizing adipokine that is involved in glucose and lipid homeostasis^33,34^. On the other hand, in adipocytes and skeletal muscle, adiponectin enhances glucose uptake via glucose transporter 4 alone or in combination with the effects of insulin^35,36^. Therefore, we hypothesized that the high adiponectin concentration may impact low blood glucose levels through increased insulin sensitivity in KO mice.

In the present study, CT promoted lipid accumulation, and physiological concentrations of CT (10^−12^ M and 10^−10^ M) suppressed adiponectin release in 3T3-L1 cells. A few studies have reported the relationship between differentiation and adiponectin production in adipocytes. Apolipoprotein E (ApoE) is highly expressed in adipose tissue and plays an important role in lipid accumulation^37^ and differentiation of adipocytes. Mice with selective suppression of adipose tissue ApoE expression have less adipose tissue and show markedly impaired triglyceride synthesis in adipocytes, but increased adiponectin levels^38^. These results reflect our present results. Brunn *et al*. have suggested that endogenous cytokines (e.g., TNF-α e.g.,interleukin-6) may inhibit adiponectin, based on the results of the inverse relationship between plasma adiponectin and cytokines *in vivo* and the cytokine-induced reduction in adiponectin mRNA in adipocyte tissue and 3T3-L1 cells^39,40^. CT may inhibit adiponectin expression in adipocytes as well as these cytokines. Further analysis of the relationship between CT and adiponectin production in adipocytes is needed.

In previous studies, we reported that a *CTR* allele is one of the genetic factors regulating human body weight^41^; this may be strongly associated with the present results. A therapeutic dose of CT reduces food intake and body weight in rats^11–13,42–44^. However, we found no significant differences in daily food intake between WT and KO mice, although KO mice had reduced body weight compared to WT in the present study. Our *in vitro* study showed that CT promoted adipocyte differentiation and that physiological concentrations of CT suppressed adiponectin release in 3T3-L1 cells. These results suggest that CT has significant effects on the regulation of metabolism, depending on its concentration and various target organs.

In the present study, old but not young KO mice showed lower weight compared to WT mice. A previous study reported that blood CT levels increase with aging in rats^45^. Genetically obese rats have unchanged or higher CT mRNA expression in comparison to lean littermates. CT mRNA is clearly higher in 10-month-old obese rats, but unchanged in 12-week-old rats^46^. Furthermore, Shiraki *et al*. reported that serum CT levels are higher in elderly human subjects with a higher body mass index^14^. These results provide strong support for our results and suggest that CT functions related to body weight may be predominantly exerted in old age.

The KO mice had significantly shorter tibial lengths than WT mice. Reduced bone growth has been reported in thyroidectomized rats^47^. From these results, it is presumed that there is some relationship between CT and bone growth. Clarifying this mechanism will likely lead to the discovery of new physiological effects of CT.

In this experimental system, the fat mass of the diet that is generally used to induce obesity is somewhat smaller, and thus, the difference in body weight in CT KO mice compared with WT mice was small. If mice are fed a high-fat diet, a greater weight difference may have occurred.

In conclusion, this is the first report in the literature to demonstrate that CT may contribute to glucose and lipid metabolism in aged male mice, possibly via decreased adiponectin secretion from adipocytes. These findings may support the therapeutic potential of targeting CT action in obesity.

## Methods

### Targeting strategy

The RENKA embryonic stem cell line was created from C57BL/6 mice and shows high rates of germline migration^48^. Mouse *Calca* (CT/CGRP) was cloned from RENKA genomic DNA and confirmed with sequencing. Exon 4 of *Calca*, which was inserted in the thymidine kinase plasmid PGKp-tk, was replaced by the neomycin resistance gene (neoR) (Fig. 1B). This targeting vector includes an 8.4-kb SpeI fragment, which serves as the 5′ targeting arm, and a 9.5-kb PvuII fragment, which functions as the 3′ targeting arm. This targeting construct was electroporated into RENKA embryonic stem cells. Neomycin-resistant colonies were selected, and embryonic stem cell clones were identified with Southern blotting (Fig. 1C). Cells from correctly targeted clones were injected into ICR blastocysts and implanted in surrogate mice. Chimeric mice were backcrossed onto the C57BL/6 background for two generations. Mice that were heterozygous for CT deficiency were interbred to generate homozygous CT^**+/+**^ (WT) and CT^**−/−**^ (KO) mice. WT littermate mice were used as controls.

### Confirmation of CT-deficient status

PCR analysis was performed to identify WT and KO mice. Common forward primers for intron 3 (int3F; 5′-TGGCACTGCCTCGCATGTC-3′) or exon 4 (exon4F; 5′-CACGTACACACAAGACCTC-3′) and reverse primers for the neomycin gene coding region (neoR; 5′-ATCTGCACGAGACTAGTGAG-3′) (KO) or exon 4 (exon4R; 5′-CTAGAAGCTCTACTAGGAAG-3′) (WT) were used. DNA was extracted from the tail using QuickGene SP Kit DNA tissue SP-DT (Kurabo Industries Ltd., Osaka, Japan) according to the manufacturer’s instructions. DNA (1 µl) was included in a 25-µl PCR mixture. Total RNA was extracted from the thyroid using the Ultraspec™ RNA isolation system (Biotecx, Houston, TX, USA) according to the manufacturer’s instructions. First strand cDNA was synthesized using 3 µg total RNA as template and oligo (dT)_12–18_ as a primer in a 20-µl reaction according to the instructions for the SuperScript™ First-Strand Synthesis System for RT-PCR (Invitrogen, Carlsbad, CA, USA). A 2-µl aliquot of cDNA was utilized in a 25-µl PCR mixture. PCR was performed using a thermal cycler (GeneAmp PCR system 9700, Tokyo, Japan) for 35 cycles. The oligonucleotide primer pairs used were CT/CGRP forward primer (exon 3 F; 5′-CTGGTGCAGGACTATATGC-3′), CT reverse primer (exon 4 R; as shown above), and CGRP reverse primer (exon 6 R; 5′-GTCACATACAACACGATGCA-3′). β-actin mRNA was detected using forward (β-ActinF; 5′-GTGGGCCGGTCTAGGCACCA-3′) and reverse (β-ActinR; 5′-GGTTGGCCTTAGGGTTCAG-3′) primers. The PCR conditions were as follows: denaturation for 30 seconds at 95 °C for all primers, annealing for 30 seconds at a temperature of 54 °C for primer pair exon4F/exon4R, or 55 °C for primer pairs int3F/exon4R, int3F/neoR, exon3F/exon4R, exon3F/exon6R, and β-actinF/β-actinR. The samples were electrophoresed using 3% agarose gels and stained with ethidium bromide.

### Mice

All animal experiments were conducted in accordance with the International Guiding Principles for Biomedical Research Involving Animals, and were approved by the Animal Care and Use Committee of Wakayama Medical University. Mice were housed under a standard light/dark cycle in facilities accredited by the Association for Assessment and Accreditation of Laboratory Animal Care, and were fed a slightly higher fat diet (7.8% fat; OA-2; CLEA Japan, Inc., Tokyo, Japan) than the standard diet (5.0% fat; CE-2; CLEA Japan). All experiments were performed using male mice.

The daily energy intake of WT and KO mice was measured at 12 weeks of age and was calculated from the daily food consumption per mouse multiplied by the gross energy for the chow of 3.58 kcal/g.

### Analysis of metabolic parameters

Mice were anesthetized with isoflurane, and whole blood was collected directly from the heart following 16 h of fasting. Serum insulin levels were measured using the Mouse Insulin ELISA kit (Mercodia AB, Uppsala, Sweden), adiponectin with the Mouse Adiponectin ELISA kit (CEMA2500-1; Assaypro LL, St. Charles, MO, USA), and TNF-α with the Quantikine ELISA Mouse TNF-α kit (R&D Systems, Minneapolis, MN, USA). Serum levels of triglyceride, NEFA (free fatty acid), and cholesterol were measured with enzyme assay kits (LabAssay Triglyceride, LabAssay NEFA, and LabAssay Cholesterol: Wako Pure Chemical Industries, Osaka, Japan). Following 16 h of fasting, blood was obtained from the tail vein, and glucose levels were measured using a portable blood glucose analyzer (Glutest Neo; Sanwa Kagaku Kenkyusho Co., LTD., Aichi, Japan).

The liver was rapidly removed, and lipids were extracted from these tissues according to the method of Folch *et al*.^49^. Briefly, snap frozen livers were homogenized and extracted with a chloroform/methanol (2:1 v/v) solution. Total cholesterol, triglyceride, and free cholesterol were measured with enzyme assay kits (CholestestCHO, CholestestTG: Shimizu Medical, Tokyo, Japan; Cholescolor LiquidFC, Toyobo, Osaka, Japan).

### ITT

The two groups of mice (WT and KO) were subjected to an ITT. Mice were allowed free access to food for 3 h and were then intraperitoneally injected with 0.75 mU/g insulin (Humulin R100; Eli Lilly Japan K.K., Kobe, Japan). Glucose levels were then measured 0, 20, 40, 60, 80, 100, 120, 140, 160, and 180 minutes later from tail vein blood using a portable blood glucose analyzer (Glutest Neo).

### Histological analysis

Mice were sacrificed under isoflurane anesthesia. The excised organs of visceral fat, liver, pancreas, spleen, and kidney were weighed. The liver, pancreas, WAT, BAT, and pancreas were fixed in 10% formalin neutral buffered solution. The fixed tissues were dehydrated, embedded in paraffin, and sectioned at 4 μm. Sections were stained with hematoxylin and eosin. Immunohistochemical staining was performed using the avidin-biotin complex method for insulin and the EnVision method for glucagon. Anti-insulin antibody (A0564; DAKO Corp., Carpinteria, CA, USA) and anti-glucagon antibody (422271; Nichirei Biosciences Inc., Tokyo, Japan) were used as primary antibodies. The antigen-antibody reaction was visualized using the chromogen 3, 3′-diaminobenzidine tetrahydrochloride. Sections were counterstained with Mayer’s hematoxylin.

### Adipocyte culture and CT treatment

The pre-adipocyte cell line 3T3-L1 (ECACC 86052701) was purchased from ECACC (Salisbury, UK). Cell culture and differentiation were performed according to a previously described protocol^50^. Briefly, Dulbecco’s modified Eagle’s medium (DMEM)-high glucose (4500 mg/L) and penicillin/streptomycin (Pen/Strep) were purchased from Life Technologies (Thermo Fisher Scientific, Darmstadt, Germany). Fetal bovine serum (FBS) was obtained from HyClone (Logan, UT, USA). Cells were grown in a 37 °C humidified environment with 5% CO_2_. 3T3-L1 pre-adipocytes were maintained in DMEM supplemented with 10% FBS and 100 U/ml Pen/Strep and allowed to reach 80% confluency. At 80% confluency, cells were harvested by trypsinization, seeded at 1.0 × 10^4^ cells/cm^2^ in 96-well microplates (Sanplatec, Osaka, Japan), and allowed to attach for 48 h. Differentiation of pre-adipocytes was subsequently induced by changing to differentiation medium, which contained 10% FBS, 100 U/ml Pen/Strep, 10 μg/ml insulin, 0.5 mM 3-isobutyl-1-methylxanthine, and 2.5 μM dexamethasone in DMEM for an additional 4 days. Thereafter, the differentiation medium was replaced with maintenance medium containing 10% FBS, 100 U/ml Pen/Strep, and 10 μg/ml insulin in DMEM. Over the next 10 days, the maintenance medium was replaced every other day to allow final differentiation of the cells. To study the effects of CT on adipocyte differentiation and production of adiponectin, different concentrations of salmon CT (0, 10^−12^, 10^−10^, 10^−8^, and 10^−6^ M; Peninsula Laboratories, Inc., Belmont, CA, USA) were added to the differentiation medium. The concentration of 10^−10^ M CT represents a physiological level^51^. Measurement of lipid contents and lipid staining in cells were done using a Lipid Assay Kit (Cosmo Bio Co., Ltd., Tokyo, Japan). Adiponectin levels in the medium were measured using a Mouse/Rat Adiponectin ELISA kit (Otsuka Pharmaceutical Co., Ltd., Tokyo, Japan).

### Statistical analysis

Data are expressed as means ± standard error of means (SEMs). Statistical analysis was conducted using JMP 11 (SAS Institute, Cary, NC, USA). All values were analyzed for normality and homogeneity of variance prior to comparisons among groups. The Shapiro-Wilk test was performed for normality, and Levene’s test was used for homogeneity of variance. The Student’s t-test was used when the assumptions of normal distribution and homogeneity of variance were met in both groups, Welch’s t test was used when the assumptions of normal distribution were met but homogeneity of variance was not met in both groups, and the Wilcoxon signed-rank test was used when the data were not normally distributed. Differences were considered significant at *p* < 0.05.
